# Development of Clindamycin-Loaded Microneedles for the Treatment of Nodular Acne: A Novel Therapeutic Approach

**DOI:** 10.1155/drp/2138049

**Published:** 2025-04-08

**Authors:** Tanikan Sangnim, Chonlada Panpipat, Supawut Chonsupawan, Siriyakorn Doungmarl, Metasit Nawayut, Kittipat Suwanpitak, Thannicha Huanbutta, Kampanart Huanbutta

**Affiliations:** ^1^Department of Pharmaceutical Technology, Faculty of Pharmaceutical Sciences, Burapha University, Muang, Chonburi, Thailand; ^2^Department of Community Health Nursing, School of Nursing, Eastern Asia University, Thanyaburi, Pathum Thani, Thailand; ^3^Department of Manufacturing Pharmacy, College of Pharmacy, Rangsit University, Muang, Pathum Thani, Thailand

**Keywords:** 3D printing, acne, microneedles, penetration, polymers, skin barrier, transdermal delivery

## Abstract

**Background:** Acne is a common and often chronic skin condition that requires prolonged treatment. Conventional topical therapies are limited by their inability to effectively penetrate the deeper layers of the skin, reducing their effectiveness in treating comedones and inflammatory acne lesions. This study aimed to fabricate dissolvable microneedles (MNs) as a novel approach for delivering clindamycin directly to the obstructed sebaceous glands beneath the skin's surface.

**Methods:** MNs were fabricated using 3D-printed molds of various shapes and lengths, employing materials such as chitosan, polyvinylpyrrolidone (PVP), and polyvinyl alcohol (PVA). Pyramid-shaped MNs, 2500 μm in length, were created using PVA soaked in sodium sulfate. Their physical properties, insertion capabilities, and dissolution profiles were evaluated through texture analysis, in vitro penetration testing, and drug release studies.

**Results:** Pyramid-shaped MNs made from PVA demonstrated the highest mechanical strength and structural integrity, confirmed through scanning electron microscopy and texture analysis. In vitro penetration testing showed that these MNs penetrated beyond four layers of Parafilm, simulating their ability to breach the stratum corneum. Dissolution studies indicated complete MN dissolution within 7–8 min, with rapid drug release occurring within 3 min.

**Conclusion:** The study demonstrates the feasibility of creating dissolvable MNs for delivering clindamycin, offering a promising alternative to conventional therapies by improving drug penetration and providing rapid drug release for the treatment of acne.

## 1. Introduction

Acne is a chronic inflammatory condition of the pilosebaceous follicle that affects individuals across various age groups, genders, and ethnicities [1, 2]. Approximately 85% of adolescents and 73% of adults aged 20 years and older experience acne at some point in their lives, with women showing a consistently higher prevalence compared to men [3, 4]. The condition arises from excessive sebum production, bacterial infection (*C. acne*), and blockage of hair follicles, leading to various types of lesions, including comedonica, papular-pustular, and nodular acne, the latter being the most severe [5–7]. Nodular acne forms deep beneath the skin and manifests as painful, scarlet bumps, often without a visible whitehead or blackhead.

Conventional topical treatments for nodular acne are limited due to poor skin permeation, necessitating the use of oral isotretinoin for severe cases. However, oral isotretinoin is associated with significant side effects, such as xeroderma, labial xerosis, photosensitivity, myalgia, and arthralgia, and is contraindicated during pregnancy [8, 9]. While transdermal approaches aim to address some of these issues, they are often ineffective for nodular acne due to insufficient drug delivery to deeper layers of the skin. The microneedle (MN) approach is novel in this regard, offering a unique ability to bypass the skin's barrier and deliver medication directly to the infected site beneath the epidermis, an advancement over existing topical and transdermal therapies [10]. Moreover, compared to oral treatment, MNs minimize systemic exposure and associated side effects, offering a safer and more targeted solution.

Clindamycin, a lincosamide antibiotic, is highly effective against *C. acnes*, the bacterial agent contributing to acne lesions. It works by inhibiting bacterial protein synthesis, thereby reducing inflammation and infection in acne-affected skin [11]. However, the efficacy of clindamycin in conventional topical formulations is hindered by poor skin permeation, leading to subtherapeutic drug concentrations in deeper layers where nodular acne resides [12]. Systemic administration, while overcoming this limitation, introduces risks of adverse effects and antibiotic resistance [13]. These challenges highlight the need for a localized delivery system capable of achieving therapeutic concentrations directly at the infection site without systemic exposure.

MNs, small needles ranging from 25 to 2000 μm in height, offer a minimally invasive alternative for transdermal drug delivery. By penetrating the stratum corneum and epidermis without reaching pain receptors or blood vessels in the dermis, MNs facilitate localized drug administration with reduced discomfort compared to conventional needles. MNs have shown potential in delivering vaccines, insulin, and other drugs [14, 15]. In this study, we hypothesize that MNs can be effectively employed to deliver clindamycin directly to the affected site in nodular acne, reducing inflammation and bacterial infection while minimizing systemic side effects.

This research focuses on developing MNs for nodular acne treatment. Chitosan, polyvinylpyrrolidone (PVP), and polyvinyl alcohol (PVA) were used to fabricate MNs, with clindamycin as the model drug. The MNs were prepared using a molding technique with 3D-printed molds of various shapes and lengths. Key parameters, including physical appearance, morphology, mechanical properties, in vitro penetration, and drug dissolution, were evaluated in this study. This study addresses a critical research gap by combining an advanced MN-based delivery platform with a proven antibiotic therapy, optimizing delivery for effective treatment of deep-seated nodular acne.

## 2. Materials and Methods

### 2.1. Materials

PVA with a molecular weight range of 85–124 kDa was procured from Sigma-Aldrich (USA). Chitosan, exhibiting a molecular weight between 30–80 kDa, was obtained from BIO21 (Thailand). Clindamycin HCl was sourced from the TS Lab (Thailand). Sodium sulfate was acquired from KEMAUS (Australia). Polyethylene glycol with a molecular weight of 10,000 was supplied by MyskinRecipes (Thailand). Silicone s812 and s814 were provided by Supersilicone (Thailand). All other chemical reagents utilized were of laboratory grade.

### 2.2. MN Design

The MNs were designed using the Shapr3D software, resulting in a computer-aided design (CAD) model. As presented in Table 1, the MNs were designed in two geometric shapes, including conical and square-based pyramids. The MN array comprises 144 needles per array for both designs. The needle length ranged from 2000 to 3000 μm. MN lengths of 2000 to 3000 μm were chosen to ensure adequate penetration depth for delivering clindamycin to the sebaceous glands, which are located 2–5 mm beneath the skin's surface [16]. Nodular acne develops due to the inflammation of sebaceous glands, where excess sebum production leads to clogged pores. The conical MN had a base diameter or diagonal of 500 μm, while the square-based pyramid MN had a base diameter or diagonal of 700 μm.

### 2.3. MN Preparation

The CAD of the MN model in the file format of stl was printed by a stereolithography (SLA) 3D printer (Original PRUSA SL1S speed, MATTERS AND FORM, Thailand) to obtain a high resolution printed product. The printing material was modified black resin. The 3D-printed MNs in different shapes were used for mold preparation. 100 g of silicone S814 was mixed with 3 g of silicone hardening agent for 3 min. Then, the mixture was poured on the 3D-printed MNs, which were placed on a plastic disc. The silicone was rested for solidification for 5 h, and then the 3D-printed MNs were removed from the mold. The MN materials were prepared using three distinct polymers: chitosan, PVA, and PVP. The amount of clindamycin (model drug) incorporated per MN array was calculated to range from 0.23 to 1.44 mg per array, depending on the MN length. This dosage exceeds the minimum inhibitory concentration (MIC_50_, 0.032; MIC_90_, 8.5) of clindamycin required for effectiveness against both gram positive (aerobes and anaerobes) and some of gram-negative (anaerobes) that cause acne inflammatory [17]. PEG 10000 was used as a backing layer in this study to provide structural support and mechanical stability to the MNs, ensuring they remain intact and functional during application. However, due to their superior mechanical properties and ease of preparation, PVA-based MNs were selected as the primary focus of this study, as highlighted in the results and discussion section. Below are the methods used to produce MNs from each type of polymer.

### 2.4. Chitosan MNs

**Figure 1:**
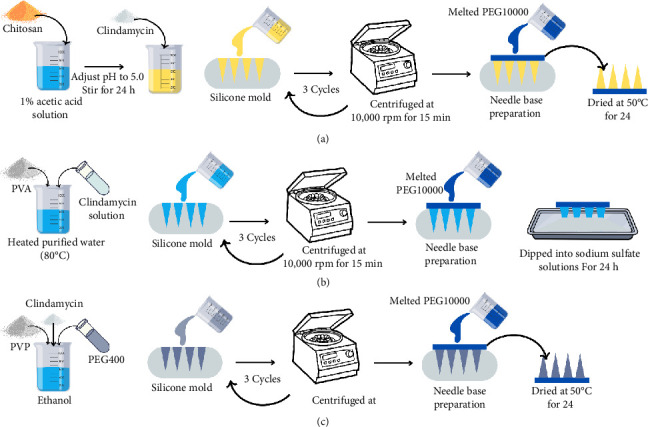
Microneedle fabrication methods prepared from (a) chitosan, (b) PVA, and (c) PVP.

To prepare chitosan-based MNs, 2 g of chitosan was dissolved in 100 mL of a 1% acetic acid solution and stirred for over 24 h as shown in Figure 1(a). The pH of the polymer solution was adjusted by adding 1 M NaOH to reach pH 5. Subsequently, 1 g of clindamycin was added and stirred until a clear solution was obtained. The chitosan with drug dispersion was transferred to a silicone mold, and it was centrifuged at 10,000 rpm for 15 min. After that, the mold containing the MN was subjected to a drying process at a temperature of 50°C for a duration of 6 h. This was followed by centrifugation and two more cycles of drying to guarantee complete filling of the mold. Subsequently, a MN foundation was fabricated by pouring molten PEG10000 into a silicone mold and allowing it to cool at ambient temperature. Afterward the MNs, along with their base, were subjected to a drying process at a temperature of 50°C for a duration of 24 h. Subsequently, the MNs were taken out from the mold.

### 2.5. PVA MNs

First, 1 g of PVA was dissolved in 10 mL of purified heat water (80°C) to enhance its solubility. Then, 1 mL of 1% (*w*/*w*) clindamycin solution was added to the PVA dispersion as presented in Figure 1(b). The PVA with drug dispersion was transferred to a silicone mold, and it was centrifuged at 10,000 rpm for 15 min. After that, the mold with the MN was dried at 50°C for 6 h, then centrifuged, and dried again for 2 additional cycles to ensure that the mold was fully filled. After that, a MN base was prepared by pouring melted PEG10000 into the silicone mold and leaving it at room temperature until it cooled. Subsequently, the MNs with a base were dried at 50°C for 24 h, and then the MNs were removed from the mold. To harden the PVA MNs, the MNs were dipped into sodium sulfate (Na_2_SO_4_) solutions at concentrations of 0.5 or 1.5 M for 24 h and dried at 50°C for 24 h.

### 2.6. PVP MNs

PVP K30 for 15 mg was dissolved in 3.4 mL of ethanol. Then, clindamycin was added to the polymer dispersion. Polyethylene glycol 400 (PEG 400) was added as a plasticizer. Then, the mixture was sonicated to enhance dissolution until the clindamycin was completely dissolved as illustrated in Figure 1(c). The PVP K30 with drug dispersion was transferred to a silicone mold, and it was centrifuged at 10,000 rpm for 15 min. Subsequently, the mold with the MN was dried at 50°C for 6 h, and it was centrifuged and dried for an additional 2 cycles to guarantee that the mold was completely filled. Afterward, an MN base was prepared by pouring melted PEG10000 into the silicone mold and allowing it to cool at ambient temperature. Afterward, the MNs with a base were desiccated at 50°C for 24 h, and the MNs were subsequently removed from the mold.

### 2.7. MN Evaluation

#### 2.7.1. Physical Appearance and Morphology

Carmoisine, a water-soluble red dye, was included into the polymer dispersion during the manufacture of the MNs to enhance the visibility of their physical appearance. Scanning electron microscopy (SEM) (LEO 1450 VP, Carl Zeiss, Germany) was employed to examine the microscopic morphology of the MN model, which offers suitable mechanical properties (high breaking force). In the sample preparation, a thin layer of 99.99% gold was applied to the MNs. Then, the MNs coated with 99.99% gold were carefully mounted onto SEM stubs using conductive adhesive to ensure proper grounding and optimal imaging conditions. Subsequently, they were placed inside the SEM chamber for high-resolution imaging and analysis of their detailed surface topography and structural integrity of the MNs.

#### 2.7.2. Mechanical Properties

The MNs, which have been removed from the mold, were evaluated for their hardness using a texture analyzer (TA.XTplusC texture analyzer, Stable Micro Systems, UK). The testing protocol involved pressing a cylindrical aluminum head with a diameter of 25 mm perpendicularly against the needles at a speed of 0.1 cm/s with a force of 0.981 N [18]. This procedure was conducted to monitor the absolute positive force making the MN break in the axial direction.

#### 2.7.3. In Vitro Penetration Test

In order to replicate the capacity of MNs to penetrate the skin, the MNs that were produced underwent evaluation using eight layers of Parafilm. This method has been adapted from a previous study conducted by Aldawood and coworkers [19]. Eight layers of blend waxes and polyolefin film (Parafilm M, Bemis, Amcor, USA), each with a thickness of 0.13 mm, were stacked to mimic the epidermal skin layer. The MNs with bases were carefully pushed until all needles were fully submerged in Parafilm. Subsequently, the MNs were removed, and the pre-existing aperture in the Parafilm within each layer was examined.

#### 2.7.4. Drug Dissolution From MN

The in vitro drug dissolution test of the MNs was evaluated by a dissolution tester (apparatus II (paddle)). The dissolution medium was 900 mL of pH 7.4 phosphate buffer solution (PBS) at a controlled temperature of 37.0 ± 0.5°C. The paddle speed was set at 100 rpm. And the medium was sampled at 3, 9, 12, and 15 min. The clindamycin amount in sampled dissolution medium was quantified by the high-performance liquid chromatography (HPLC) method (SPD-M20A, Shimadzu, Japan). The method was applied to the previous study by Kampanart and team [20]. The stationary phase was a reverse phase C18 column (25 cm × 4.6 mm, 5 μm) (ACE5C18, V17-1586, UK) maintained at a temperature of 40°C. The mobile phase was a mixture of acetonitrile and pH 7.5 phosphate buffer in a ratio of 9:11 (*v*/*v*), with an isocratic flow rate of 1.0 mL/min.

### 2.8. Statistical Analysis

The data acquired from distinct experiments underwent statistical analysis utilizing descriptive statistics. Subsequently, for comparative analysis of the data, Student's *t*-test was employed to ascertain any significant differences between the groups.

## 3. Results and Disscussion

### 3.1. Physical Appearance and Morphology

MN-forming materials are crucial for determining their physical characteristics. The chitosan MN exhibited characteristics of being thin, pliable, and supple. The PVP-based materials had a partially solid texture, did not harden, and were unable to stick to the base for easy removal from the mold. The MNs, composed of PVA, exhibited a sharp, robust, and pliable nature. Subsequently, when PVA MNs were submerged in sodium sulfate and subjected to a drying process at a temperature of 50°C for a duration of 24 h, the MNs exhibited enhanced sharpness, strength, and improved ease of removal from the mold. This enhancement is likely attributed to cross-linking induced by sodium sulfate, which forms covalent bonds between PVA molecules. This cross-linking process results in a more rigid and structured material, improving the MNs' mechanical properties [21]. While chitosan and PVP were found unsuitable in this study due to their limitations in formability and mechanical properties of MN [22], this may be attributed to the need for selecting appropriate polymer grades and types. Additionally, the incorporation of additives, such as crosslinkers and plasticizers, has the potential to enhance the mechanical properties of MNs [22].

The characteristics of MNs varied in size and shape. Conical-shaped needles and square pyramid-shaped needles were prepared by using PVA as the needle-forming material. Conical-shaped needles appeared uniformly strong and sharp but had fewer needles adhering to the base compared to square, pyramid-shaped needles. Square pyramid-shaped needles appeared relatively strong; however, some needles were incompletely and inconsistently filled with PVA. The MNs with a length of 2000 μm were not effectively fabricated due to the challenge of detaching them from the mold. The MNs' length ranging from 2500 to 3000 μm exhibited no discernible differences in terms of their appearance. Thus, MNs with a conical form and a specific length were chosen for subsequent testing and development. The demolding difficulty observed in our research could be attributed to several factors. A primary reason may be the adhesion between the hydrophilic polymers (PVP, PVA, and chitosan) and the silicone mold. Since these polymers are highly hydrophilic, they may form stronger interactions with the mold surface, increasing friction and making the demolding process more challenging [23]. To address this issue, further formulation development or the selection of appropriate grades and types of polymers is necessary to optimize the MN preparation process. Additionally, using different materials for the MN base and the MNs themselves might facilitate easier detachment compared to using similar materials. Lastly, the geometry and design of the MNs might also affect the demolding process [24–26].

**Figure 2:**
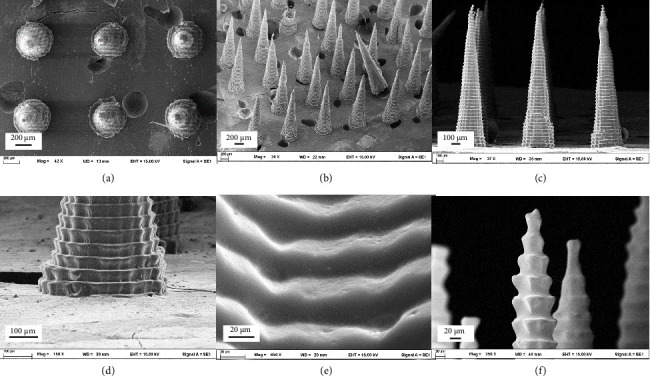
SEM images of PVA microneedles from various perspectives, including (a) top view, (b) oblique view, (c) side view, (d) basement, (e) surface, and (f) tip.

SEM images of PVA MNs in cone-shape design from various perspectives are presented in Figure 2. From different angles of SEM images (Figures 2(a), 2(b) and 2(c)), MNs were formed uniformly in a conical shape with a tapering and layered structure. Focused on the base (Figure 2(d)), the needles were fully attached to the basement, representing a potential connection between two polymers, which were PVA (MN) and PEG10000 (basement). PEG10000 was selected as the base layer for the MNs due to its favorable physical and mechanical properties. Since the MN material must dissolve within the skin layer, its mechanical properties alone may not be sufficient to ensure stability during handling, transportation, and application (e.g., pressing onto the skin). Additionally, using the same material for both the base and the MNs could make it difficult to detach the MNs from the base, which is essential for effective application and retention of the MNs in the skin. Introducing PEG10000 as a separate base layer addresses this issue by providing structural support while allowing for easy detachment of the MNs. Furthermore, PEG10000 enhances the overall stability of the MNs and helps prevent premature detachment from the base during storage or transportation. As shown in Figure 2(e), the surface of the MN was undulating from the layered structure. This wavy surface is likely attributed to the mold, which was prepared using a 3D-printed MN prototype. Despite utilizing the SLA 3D printing technique, the resolution of the printed prototype was not sufficiently high [27]. As SLA printing is a layer-by-layer process, this likely contributed to the wavy surface of the MN prototype used to create the mold. The tip of the MNs had sharpness and fine structural details essential for effective skin penetration, as illustrated in Figure 2(f). The reason why the MN structure was in a layer form was because the mold was prepared by an SLA 3D printer that has a printing pattern in the format of layer by layer. Even though SLA has better printing resolution, especially for rounded elements, than the fuse deposition modeling (FDM) printing technique, the desktop SLA printer could not print a flat surface at the microscopic level for very small objects such as MNs. This made the printed MNs slightly different from the CAD model [28, 29].

### 3.2. Mechanical Properties

**Figure 3:**
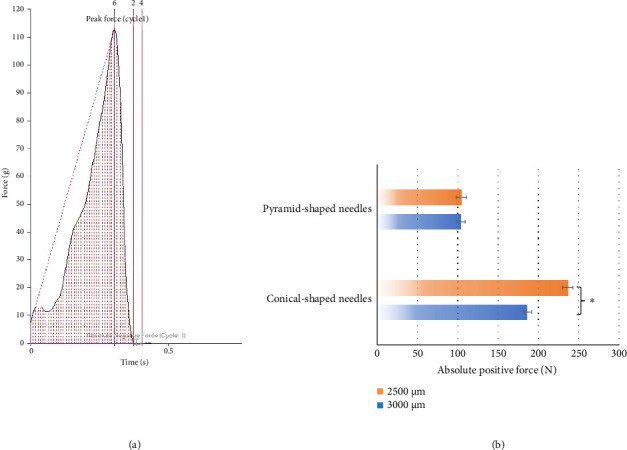
(a) Mechanical property hardness profile of the PVA microneedle and (b) breaking force of PVA microneedles in different shapes and lengths (⁣^∗^a significant difference at a *p* value of less than 0.05).

PVA-based MNs were selected for the mechanical property test in this study due to their ease of preparation. The mechanical properties of PVA MNs in different shapes (conical and square-pyramid) and lengths (2500 and 3000 μm) were measured in terms of breaking force which is the absolute positive force at the peak of the hardness profile, as illustrated in Figure 3(a). Figure 3(b) displays the maximum positive force at the highest point of the hardness profile of MNs with two different shapes and lengths. The breaking force of MNs in shape was around 104 N, which was significantly lower than that of conical-shaped MNs. The reason for this could be attributed to the conical structure providing equal force distribution along the whole needle. This make structure of the conical needle can resist breaking force from axial direction. Comparing to the square pyramid shape MN, this structure is more susceptible to fracturing when subjected to lesser forces [30]. Moreover, Figure 3(b) also demonstrates that the shorter needle (2500 μm) of conical-shaped MNs offered greater strength compared to the longer one (3000 μm). This is likely due to the elongation of the MN, which increases the potential for structural bending and buckling pressures. These pressures result in an intensified stress concentration, leading to a higher likelihood of flaws in the MN material, ultimately weakening its mechanical strength. The findings suggest that shorter MNs may provide better mechanical stability, making them more suitable for applications requiring higher penetration forces [30]. A previous study by Davis and colleagues reported that across different MN geometries, insertion force varied linearly with the interfacial area of the needle tip. Measured insertion forces ranged from approximately 0.1–3 N, which is sufficiently low to permit insertion by the hand [31]. Therefore, the mechanical testing demonstrated that the MNs maintained adequate strength and structural integrity.

### 3.3. In Vitro Penetration Test

**Figure 4:**
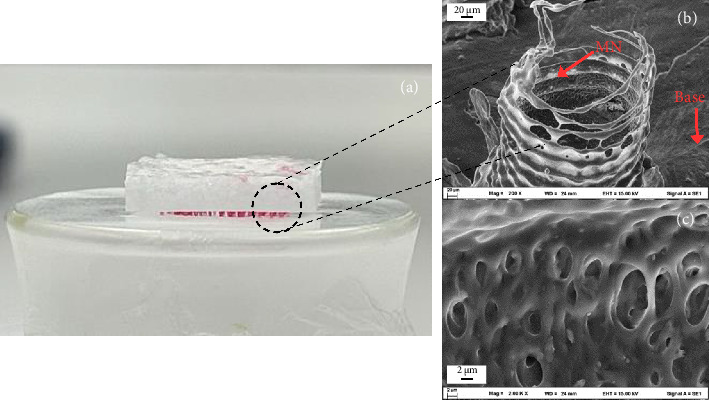
In vitro penetration test of (a) microneedles (MN) penetrating a eight-layer stack of Parafilm. Scanning electron microscope (SEM) images of the microneedle: (b) base and (c) surface.

MNs fabricated from PVA, shaped as cones and pyramids with a square base and a length of 2500 μm, successfully penetrated all four layers of Parafilm with a total thickness of 0.5 mm, as shown in Figure 4(a). The results showed that most MNs in the array successfully penetrated through 4 layers of Parafilm, indicating an estimated penetration depth of 508 μm. This depth is hypothetically sufficient to reach deep into the epidermis layer after human skin penetration [32]. Furthermore, some MNs penetrated through all 8 layers of Parafilm (1016 μm depth), suggesting that the MNs can potentially target sebaceous glands located in the dermis, the second layer of skin, which are typically about 1 mm deep [33]. SEM images were employed to investigate the mechanisms of MN detachment (Figures 4(b) and 4(c)). A hollow structure was discovered between the needle and the base (Figure 4(b)). This might be because using a different material for the backing layer (PEG1000) allows the MNs to detach more easily from the base after pressing and removing the MN patch from the skin. Additionally, the MN was exceedingly thin. These facilitate the detachment of MNs following needle penetration. For the treatment of acne, it is advantageous to maintain the MN in the epidermis to facilitate drug release and polymer dissolution, which leads to a more effective therapeutic outcome [34].

While the use of Parafilm in this in vitro penetration test represents a valid initial approach for evaluating MN efficacy, it is important to acknowledge that Parafilm does not fully replicate the mechanical properties of actual skin. Specifically, it lacks the elasticity, viscoelastic response, and hydration characteristics inherent to human skin. Consequently, results obtained from Parafilm penetration studies may not be directly translatable to clinical outcomes. Therefore, future studies should incorporate penetration tests using more representative ex vivo or in vivo skin models to ensure more accurate and clinically relevant findings.

### 3.4. Drug Dissolution From MN

**Figure 5:**
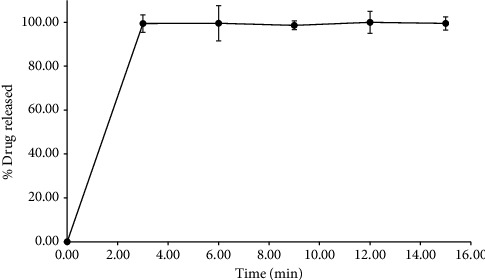
Dissolution profile of clindamycin HCl from microneedles prepared from PVA.

Figure 5 illustrates the drug dissolution profile of clindamycin that is dissolved from MNs. Clindamycin demonstrated a rapid initial explosive release, reaching nearly 100% within the first 3 minutes, as was observed. This was followed by a plateau of 100% drug release for 15 min, which suggested that the MNs had been completely dissolved. The rapid drug release is facilitated by the high surface area-to-volume ratio of the MN surface and dissolution medium, as indicated by the Noyes–Whitney equation [35]. Furthermore, clindamycin hydrochloride is classified as a biopharmaceutics classification system (BCS) Class III drug, which is distinguished by its high solubility, which further contributes to its rapid dissolution profile [36]. The rapid dissolution of the MNs minimizes the duration of skin contact, thereby enhancing patient comfort. Moreover, clindamycin's antibacterial mechanism is concentration-dependent. Prolonged MN insertion may increase the risk of skin irritation, making fast drug release more suitable for this application. Consequently, extended drug release may not be advantageous in this particular case [37–39]. However, it is important to note that the drug dissolution test performed using Dissolution Apparatus II may not directly mimic the conditions of drug release from MNs in human skin. Therefore, further in vitro and in vivo studies are needed to evaluate the dissolution behavior of clindamycin under more clinically relevant conditions.

## 4. Conclusion

The PVA-based MNs were successfully fabricated using a molding technique that replicated those prepared by SLA 3D printing. Dipping the PVA MNs in sodium sulfate for 24 h enhanced their physical characteristics, resulting in improved mechanical strength and structural integrity. The cone-shaped MNs, with a length of 2500 μm, exhibited robust mechanical properties, as demonstrated by their successful penetration through four layers of Parafilm in in vitro penetration tests. This suggests their potential for delivering drugs through the stratum corneum for the treatment of nodular acne, which occurs beneath the skin surface. Additionally, SEM analysis showed that the MNs detached from the base after insertion, and they demonstrated rapid drug release within 3 min, a desirable property for dissolvable MNs in transdermal drug delivery. These results highlight the potential of PVA-based MNs for nodular acne treatment. However, to address the challenges associated with clinical applications, additional considerations are necessary. While these MNs exhibit promising mechanical and drug release properties, potential issues such as skin irritation, manufacturing scalability, and regulatory hurdles must be carefully evaluated. Clinical experiments are necessary to confirm the ability of these MNs to penetrate beyond the stratum corneum to the subcutaneous layer effectively. Future research should focus on evaluating the performance of these MNs in therapeutic models, such as ex vivo pig skin or in vivo mouse models, to further validate their clinical applicability. In addition, the scalability of the fabrication process should be explored to assess feasibility for large-scale production. Addressing regulatory requirements and ensuring compliance with clinical safety standards will be critical steps for translating this technology into practical therapeutic applications.

## Figures and Tables

**Table 1:** Microneedle design information.

Microneedle design	Shape	Length (μm)	Base diameter or diagonal of the base (μm)
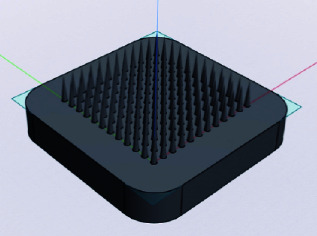	Conical	2000	500
		2500	500
		3000	500

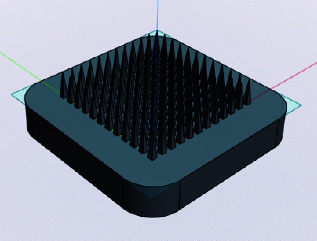	Square-based pyramid	2000	700
		2500	700
		3000	700

## Data Availability

The data that support the findings of this study are available from the corresponding author upon reasonable request.
